# Acute and Subacute Toxicity Profiles of the Methanol Extract of *Lycopersicon esculentum* L. Leaves (Tomato), a Botanical with Promising *In Vitro* Anticancer Potential

**DOI:** 10.1155/2020/8935897

**Published:** 2020-03-05

**Authors:** Gaëlle S. Nguenang, Arsène S. M. Ntyam, Victor Kuete

**Affiliations:** Department of Biochemistry, Faculty of Science, University of Dschang, Dschang, Cameroon

## Abstract

*Lycopersicon esculentum* (tomato) is a plant widely used in Africa like food and to solve many health problems. The methanol crude extract of tomato recently demonstrated a good antiproliferative effect on many human cancer cell lines. The aim of this research was to evaluate the acute toxicity and subacute oral toxicity of methanolic extract from leaves of this plant. These toxicities were evaluated based on the OECD (Organization for Economic Cooperation and Development) guidelines. The assay of acute toxicity was performed using a total of 3 female rats, which received a single dose of 5000 mg/kg of methanolic extract via oral gavage. For the subacute toxicity study, 32 *Wistar* rats (males and females) were used. The groups were treated with three different doses of *Lycopersicon esculentum* methanolic extract (250, 500, and 1000 mg/kg b.w.) for 28 days and the control group received distilled water. The hematological, biochemical, and histopathological studies were performed after the sacrifice. Single dose of tomato extract caused no toxicity up to a dose of 5000 mg/kg body weight; hence, the median lethal dose (DL_50_) of leaves of this plant was greater than this value. However, lower toxic effects could be manifested in the long-term treatment at the highest dose (1000 mg/kg) because urea level and total serum proteins significantly increased at a dose of 1000 mg/kg with respect to control. The microscopic observation showed no remarkable pathological changes on all organs in the treated groups compared with the control groups of female and male rats. These results demonstrate that single dose of tomato extract leaves is relatively nontoxic at a dose of 5000 mg/kg b.w. and prolonged use of lower doses (250 and 500 mg/kg) of *L. esculentum* orally should be encouraged, whereas highest dose (1000 mg/kg) should be avoided.

## 1. Introduction

Phytochemicals have beneficial effects on health when consumed by humans and can be used to effectively treat human diseases [1, 2]. According to the World Health Organization (WHO), about 40–90% of people living in developing countries frequently use traditional medicine [3] for their primary health care and almost three-fourths of the herbal drugs used worldwide are derived from medicinal plants [4]. Studies conducted by Boumediou and Addoun (2017) revealed that out of 80 plant species identified, 9 were toxic and 21 were slightly toxic [5]. More experimental data on the toxicity profile of medicinal plants and their extracts are essential to increase human safety and their use in the development of pharmaceuticals [6]. Therefore, the evaluation of potential toxicity of medicinal plants is a necessary step for the validation of their regular therapeutic use [7].

Tomato widely called *Lycopersicon esculentum* L. belongs to the family of *Solanaceae* [8]. It is a native plant of west coast of South America, which is mostly well-liked and commonly grown vegetable all over the world [9].This plant is used in traditional medicine as anti-inflammatory and antimicrobial and to treat heart diseases, age-related diseases [10], and cancer [11]. The leaves of chopped tomatoes are applied to the skin as a remedy for insect bites [12]. In tomato, both leaves and fruits contain secondary metabolites, which protect hosts against adverse effects of predators including fungi, bacteria, viruses, and insects involved in host-plant resistance [12, 13].

Previous phytochemical investigations of this plant led to the isolation of lycopene, *β*-carotene, lutein, zeaxanthin, flavonoids, hydroxycinnamic acid, and glycosides [14]. However, Mbaveng et al. showed that the leaves of this plant had cytotoxic properties [11]. In effect, it was shown that the leaves of this plant had a wide range of cytotoxic activity toward both hematological and carcinoma cell lines, including drug-sensitive and multidrug-resistant phenotypes; these cancer cell lines included CCRF-CEM leukemia cells (IC_50_ value of 17.94 *μ*g/mL), its resistant subline CEM/ADR5000 cell line (IC_50_ value of 37.70 *μ*g/mL), the MDA-MB-231-pcDNA breast cancer cell line (IC_50_ value of 9.64 *μ*g/mL) and its resistant counterpart MDA-MB-231-BCRP (IC_50_ value of 11.94 *μ*g/mL), the HCT116 (p53^+/+^) colon cancer cell line (IC_50_ value of 15.37 *μ*g/mL) and its resistant counterpart HCT116 (p53−/−) (IC_50_ value of 14.89 *μ*g/mL), the U87MG glioblastoma cell line (IC_50_ value of 14.89 *μ*g/mL) and its resistant subline U87MG.ΔEGFR (IC_50_ value of 18.76 *μ*g/mL), and the HepG2 hepatocarcinoma cell line (IC_50_ value of 59.94 *μ*g/mL) [11]. Furthermore, Manekeng et al. had demonstrated that tomato leaves extract inhibits the growth of Gram-positive *Staphylococcus* bacteria [15].

Several epidemiological studies have shown that tomato consumption is beneficial in the cancer and cardiovascular disease prevention [12]. This plant is known to be associated with a reduced risk of developing some chronic diseases and acts as an antioxidant due to the presence of lycopene, a bioactive carotenoid [16, 17]. It has been reported that dried tomato pomace exhibited cognitive enhancing effect in normal and cognitive impairment conditions [18]. *L. esculentum* L. contains nutrients that prevent illnesses by detoxification [19, 20], promoting growth [21], and proper immune system functioning [22], as well as increasing the hematocrit, red blood cell, and white blood cell content [23].

However, the harmful effects of tomatine (tomato glycoalkaloids) are to disrupt cellular membranes [24–26] and to inhibit acetylcholinesterase and butyrylcholinesterase activities like other glycoalkaloids such as *α*-solanine and *α*-chaconine [25, 27]. Knowing the potential harmful effect of this plant lad us to believe that tomato leaves could be toxic for the human body; hence, performing toxicological studies of this plant become imperative given that little information about tomato leaves is known. The aim of this study was to evaluate the acute and subacute toxicities of tomato leaves through oral administration.

## 2. Material and Methods

### 2.1. Plant Material

Fresh leaves of this plant were harvested on April 2018 at BIOMAR-Cameroun (experimental biological field) located in Dschang, in the Menoua division of the West Region of Cameroon. The identification of this plant was made at the National Herbarium of Cameroon under the number 43088/HNC.

### 2.2. Preparation of Crude Methanolic Extract

In order to obtain the Cameroonian food plant extract, the harvested leaves were cleaned, dried, and ground. The resulting powder was macerated in methanol in 1:3 proportion at room temperature. The mixture was stirred around 3 to 4 times per day, in order to maximize the yield. After 48 hours, it was filtered through Wattman filter paper (no.1), and the filtrate was evaporated using rotary evaporator (Buchi R-200) at 65°C. The crude extract was collected in sterile flask and dried by oven (40°C); the yield of plant extract was calculated in relation to the powder mass of the dry plant.

### 2.3. Experimental Animals

Adult *Wistar* rats (09 to 11 weeks old) were used for the acute and subacute toxicity studies. These animals were raised at the animal house, Department of Biochemistry, University of Dschang, where they were fed by a standard rat diet and had free access to water. They were maintained at standard laboratory conditions of regular 12 h light/12 h dark cycle and temperature (24 ± 1°C) throughout the experimental period. Animals were acclimatized for a week before each of the experiments.

### 2.4. Acute Oral Toxicity Study

The assay of acute toxicity was performed according to the OECD guidelines No. 425 [28]. A total of 3 nulliparous and nonpregnant female rats (aged 9-10 weeks) were used, and each rat received a plant extract. After 12 hrs starvation (food suppressed, but not water), the dose of 5000 mg/kg was administered by gavage using an endogastric tube. The animals were regularly and individually observed for behavioural and general toxicity signs after dosing for the first 24 hrs, with special attention being given during the first 4 hrs. Thereafter, observation was continued daily for a total of 14 days. On the 15th day, the rats were starved overnight; the measurement of body weight of rats was performed, and vital organs (liver, heart, kidneys, lung, and spleen) were removed for macroscopic examination.

### 2.5. Subacute Oral Toxicity Study

The subacute toxicity study was conducted in compliance to the OECD Guidelines No. 407 [29]. 32 male and female *Wistar* rats, including 16 males and 16 females, aged from 09 to 11 weeks were randomly distributed for each sex in 4 groups of 4 rats per group. The groups were treated with three doses of *L. esculentum* methanolic extract (250, 500, and 1000 mg/kg b.w.) for 28 days, and the control group received distilled water. The animals were regularly and individually observed for behavioural changes and general toxicity signs after dosing at the end of each day. During the experimental period, the body weights of all groups were measured after every four days. At the end of the treatment period, all rats fasted overnight (12 h). Blood samples were collected for the measurement of hematological (EDTA coated tubes) and biochemical (dry tubes) parameters. Organs (heart, kidneys, lung, spleen, and liver) were removed for weight measurement and histopathological examination. The relative organ weight (ROW) of each animal was then calculated as follows: ROW = [Absolute organ weight (g) ÷ Bodyweight of rat on sacrifice day (g)] × 100.

### 2.6. Hematological Parameters

For the hematological parameters, all animals were starved during the night, and the blood sample was collected by cardiac puncture after sacrifice. This blood was stored in the EDTA tubes, and the hematological analysis was performed using an automated analyzer hematology (QBC Autoread plus, United Kingdom). Parameters evaluated included white blood cells (WBCs), lymphocytes (LYMs), red blood cells (RBCs), hemoglobin (Hb), hematocrit (HCT), mean corpuscular volume (MCV), mean corpuscular hemoglobin (MCH), mean corpuscular hemoglobin concentration (MCHC), platelets (PLTs), monocytes, granulocytes, and mean platelet volume (MPV).

### 2.7. Biochemical Parameters

For the measurement of biochemical parameters, dry tubes containing collected blood were centrifuged at 3000 rpm for 15 min to obtain the serum. Diagnostic kits (SGMitalia) were used to evaluate the following parameters: total serum protein (TP), alanine aminotransferase (ALT), aspartate aminotransferase (AST), serum urea (UREA), serum creatinine (CREA), low-density lipoprotein-cholesterol (LDL-C), high-density lipoprotein-cholesterol (HDL-C), total cholesterol (TC), and triglycerides (TG).

### 2.8. Histopathological Examination

Liver and kidneys were dissected and rinsed in saline solution before their weights were measured. These tissues previously preserved in 10% formalin were dehydrated in a graded series of ethanol and enclosed in paraffin. Thereafter, 5 *μ*m sections were prepared using a microtome and stained with hematoxylin-eosin prior to microscopic examination. The microscopic features of the organs of treated groups were compared with that of the control group [30].

### 2.9. Statistical Analysis

The results expressed as mean ±  standard deviation have been submitted to the analysis of variance (ANOVA) at one factor according to the general linear model. Statistical analysis was performed using version 21 of the IBM-SPSS statistical program, and statistical comparisons were made using the test of Waller–Duncan for the subacute toxicity at the 5% probability level.

## 3. Results

### 3.1. Acute Oral Toxicity

During the acute toxicity, no animal death was registered. All female rats received 5000 mg/kg of *L. esculentum* methanolic extract. No sign of toxicity was observed in the behaviour of rats during the 14-day observation period. Therefore, the approximate acute lethal dose (LD_50_) of this extract in female rats was estimated to be higher than 5000 mg/kg. Tables 1 and 2 contain the body weights (g) and relative organ weights in the female rats in acute toxicity, respectively.

### 3.2. Subacute Oral Toxicity

#### 3.2.1. Effect of Oral Administration of L. Esculentum Extract on Food Consumption

**Figure 1:**
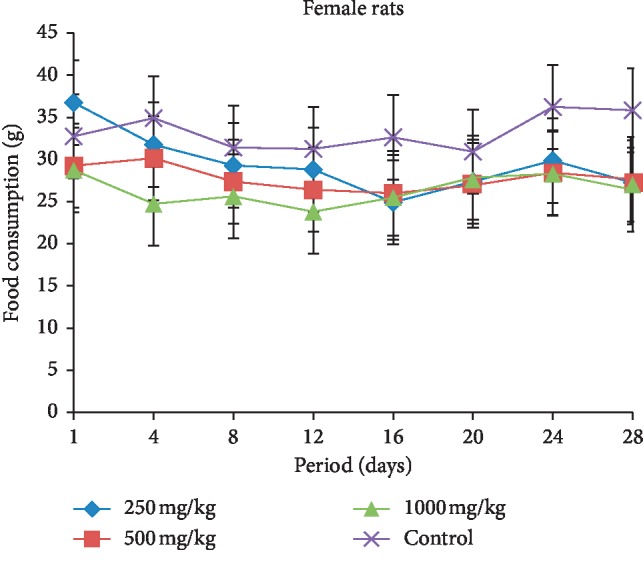
Evolution of food consumption according to the dose extract during the treatment in the female rats.

**Figure 2:**
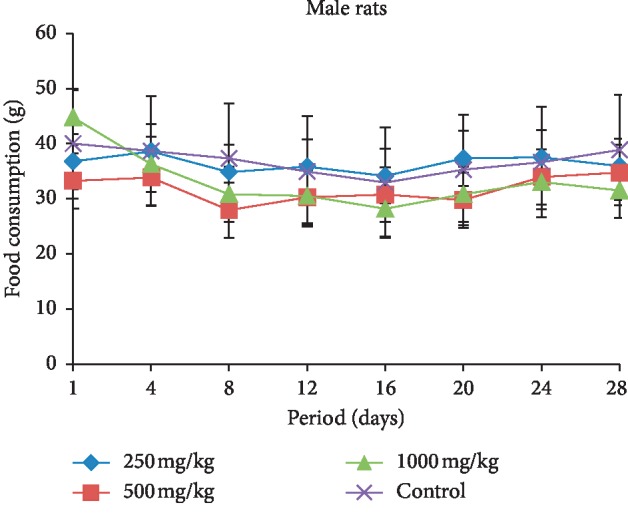
Evolution of food consumption according to the dose extract during the treatment in the male rats.

The food consumption in both female and male rats treated with different doses (250, 500, and 1000 mg/kg b.w.) of extract is presented in Figures 1 and 2. In general, the female and male rats showed decrease in the food consumption compared with the control groups during the treatment period. However, the reduction of food intake in female groups was significant from the 22^nd^ day of treatment with respect to the control group.

#### 3.2.2. Effect of Oral Administration of L. Esculentum Extract on Body Weight

**Figure 3:**
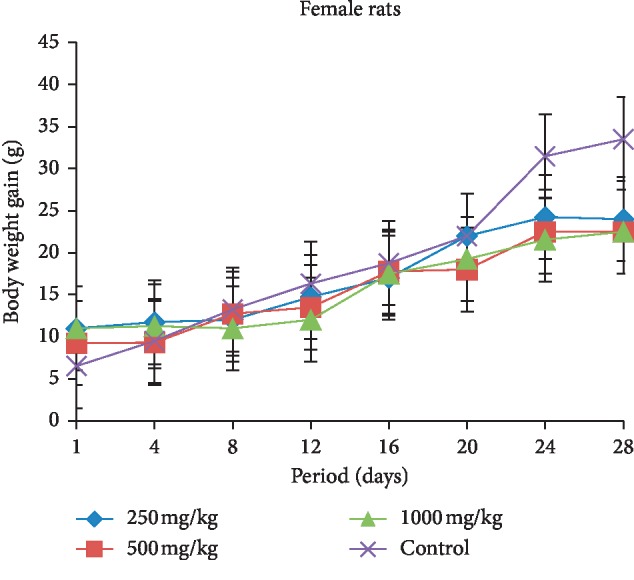
Evolution of body weight according to the dose of extract during the treatment in the female rats.

**Figure 4:**
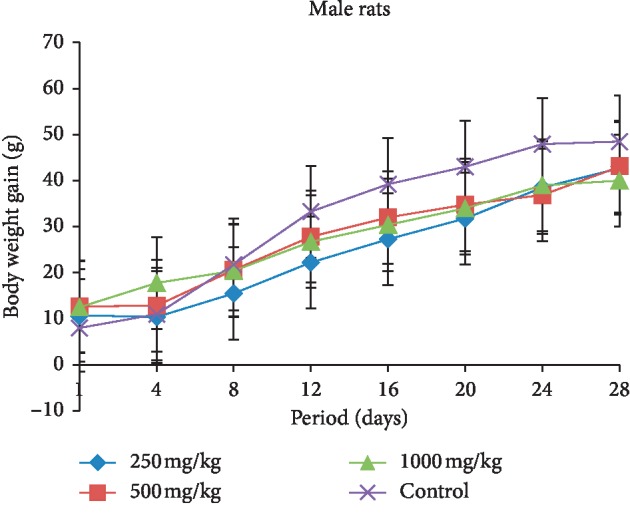
Evolution of body weight according to the dose of extract during the treatment in the male rats.

The body weight gain in both female and male rats treated with different doses (250, 500, and 1000 mg/kg b.w.) of extract is presented in Figures 3 and 4. The female and male rats showed a decrease in their body weight compared with the control group during the treatment period. In the female rats, the body weight gain reduced inversely proportional to doses administered. This body weight of treated groups reduced significantly compared with the control group from the 22^nd^ day of treatment, and the same effect was produced between the 12^th^ day and the 24^th^ day of treatment in male rats. Moreover, after 28 days of treatment, the male rats that received 250 mg/kg extract showed more weight gain than other treated rats compared with the beginning of treatment.

#### 3.2.3. Effect of Oral Administration of Leaves of L. Esculentum Extract on Organ Weights

Table 3 presents the results of the relative organ weights after the exposure of both female and male rats to the methanolic extract of *L. esculentum*. The results showed that no significant difference (*p* < 0.05) was observed in organ weights of treated rats compared with the control rats.

#### 3.2.4. Effect of Oral Administration of Extract on Serum Transaminases (ALAT and ASAT) Activity and Total Serum Protein Level

The effect of different doses of extract on the activity of serum transaminases is shown in Table 4. The results indicate that in male rats, after repeated administration doses of extract, the activity of serum transaminases (ALAT and ASAT) was significantly decreased (*p* < 0.05) compared with the control. In the female rats, no significant difference was observed in the activity of ALAT. Nevertheless, the activity of ASAT was significantly decreased at the doses of 250 and 1000 mg/kg compared with the control group.

The level of serum total proteins showed no significant difference in both sexes compared with the control groups except at the dose of 1000 mg/kg in male rats, where this level of proteins showed significant increase levels compared with the control.

#### 3.2.5. Effect of Oral Administration of Extract on Serum Urea and Serum Creatinine

Table 5 presents the effect of *L. esculentum* extract in the level of serum creatinine and serum urea. After analysis, creatinine level was not significant both in male and female rats compared with their control groups. Urea level was not significant at doses of 250 and 500 mg/kg but significantly increased at a dose of 1000 mg/kg in both sexes with respect to control.

#### 3.2.6. Effect of Oral Administration of L. Esculentum Extract on Serum Lipid Profile

Table 6 presents effect of administration of methanolic extract of the leaves of *L. esculentum* on lipid profile in both female and male rats. An increment in high-density lipoproteins level (HDL) was observed in female rats treated at doses of 250 and 500 mg/kg of extract compared with their control group. However, the level of atherosclerosis index was significantly lower in doses of 250 and 500 mg/kg compared with control. In male, a significant decrease in triglyceride levels and total cholesterol was observed in rat groups treated with extract at all doses; similarly, the level of atherosclerosis index was significantly lower in doses of 250 and 1000 mg/kg.

#### 3.2.7. Effect of Oral Administration of L. Esculentum Extract on Hematological Parameters

Hematological parameters of female and male rats were examined as shown in Table 7. Significant reduction was observed in the level of platelets in the female animals treated with extract of *L. esculentum* compared with controls. In the male rats, WBCs, lymphocytes, and platelets were significantly higher at all doses compared with control group. Other hematological parameters measured did not show significant differences compared with the control groups.

### 3.3. Histopathological Examination

**Figure 5:**
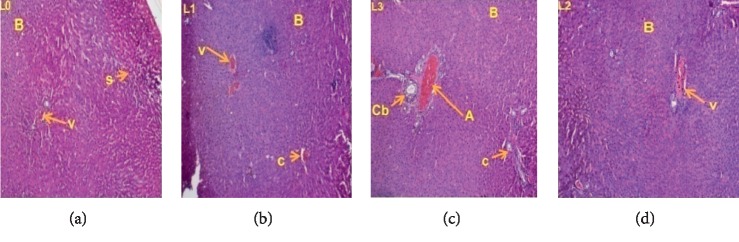
Liver sections showing the effect of *L. esculentum* methanolic extract in 28-day subacute toxicity study in female rats. (a) Dose 0 mg/kg. (b) Dose 250 mg/kg. (c) Dose 500 mg/kg. (d) Dose 1000 mg/kg.

**Figure 6:**
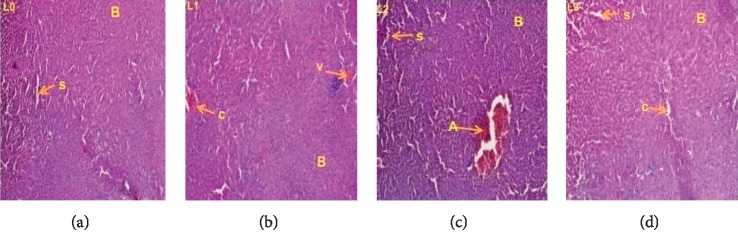
Liver sections showing the effect of *L. esculentum* methanolic extract in 28-day subacute toxicity study in male rats: (*L*_0_): control group; (*L*_1_): 250 mg/kg; (*L*_2_): 500 mg/kg and (*L*_3_): 1000 mg/kg. Indicators: (A): hepatic portal vein; (B): hepatocytes; (V): centrolobular vein; (C): leukocyte infiltration (inflammation); (S): sinusoid; (Cb): bile duct. (a) Dose 0 mg/kg. (b) Dose 250 mg/kg. (c) Dose 500 mg/kg. (d) Dose 1000 mg/kg.

**Figure 7:**
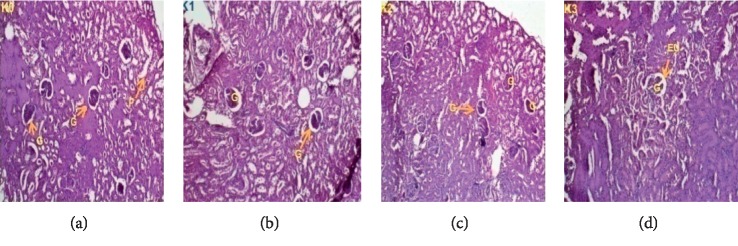
Kidney sections showing the effect of *L. esculentum* methanolic extract in 28-day subacute toxicity study in female rats.

**Figure 8:**
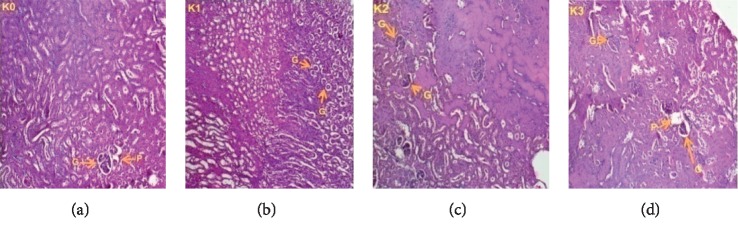
Kidney sections showing the effect of *L. esculentum* methanolic extract in 28-day subacute toxicity study in male rats:(*k*_0_): control group; (*k*_1_): 250 mg/kg; (*k*_2_): 500 mg/kg; and (*k*_3_): 1000 mg/kg. Indicators:(G): glomerulus; (EU): urinary tract; (A): distal tubule; (P): proximal tubule. (a) Dose 0 mg/kg. (b) Dose 250 mg/kg. (c) Dose 500 mg/kg. (d) Dose 1000 mg/kg.

Histopathological examinations were performed on the liver and kidneys to assess whether these organs or tissues had been damaged. The microscopic observation showed no remarkable pathological changes on all organs in extract treated groups compared with the control groups of female (Figures 5 and 6) and male rats (Figures 7 and 8).

## 4. Discussion

In the present study, single oral dose (5000 mg/kg b.w.) of extract of *L. esculentum* administered in female rats had no effect on mortality and examined clinical signs. Therefore, no acute toxicity was found in rats treated with this extract, and approximate LD50 was determined to be higher than the dose of 5000 mg/kg. Yet the lack of toxicity-indicative manifestation suggests that the administration of these leaves can be attributed to subsufficient absorption of the extract or component of extract in the gastrointestinal tract or a high first-pass metabolism in the liver, by which toxic components would have been converted to their harmless derivatives. This first hypothesis is in harmony with the results of Mendel (2002), which showed that the absorption of tomatine from gastrointestinal tract is poor because when it is orally ingested, much of them may form complexes with cholesterol from the other food present in the stomach [13]. These complexes are not absorbed in the intestine but are excreted [13]. Nonetheless, the knowledge gained from our acute toxicity study may serve as a base for choosing more appropriately the dose of *L. esculentum* for subacute toxicity study.

The subacute toxicity study, which involved rats given the extract of *L. esculentum* at doses of 250, 500, and 1000 mg/kg, demonstrated that the food consumption and animal growth change concomitantly, which means a direct effect of food consumption on weight loss or gain in animals. Daily administration of the different doses caused a decrease in food intake and in animal growth compared with the control rats in both sexes. These significant reductions from the 22^nd^ day of treatment in the female rats treated may be due to the fact that tomatine contained in the plant extract caused abdominal pain in animals that would prevent rats from eating well and therefore loss more weight. The symptoms of acute tomatine poisoning in animals are similar to that of poisoning by solanine, a potato glycoalkaloid [31–33]. These clinical symptoms include gastrointestinal and neurological symptoms, particularly vomiting, headache, and flushing [34]. Studies have shown that the administration of high concentrations can affect intestinal function [35]. However, McMillan and Thompson proved that this glycoalkaloid (solanine) content of young leaves is substantially higher than the tubers in one *Solanaceae* of the genus *Solanum* (*S. tuberosum*). Therefore, this might lead to believe that the ingestion of a large amount of plant leaves of this genus can cause gastrointestinal distress [36].

The liver is the central organ of metabolism. In case of liver damage, we observe an increase in transaminase activity (ALT and AST), and when the damage increases, this activity also increases [37]. Furthermore, when liver cell membrane is damaged, a variety of enzymes normally located in the cytosol are released into the blood stream. Measuring the activities of serum marker enzymes like ALT, AST, and ALP, as well as level of serum total bilirubin has provided a powerful tool for the assessment of liver function [38, 39]. However, the reduction of serum transaminase levels in male (at all doses) and female (ASAT, doses 250 and 1000 mg/kg) rats compared with the control observed in this work could indicate that the methanolic extract of *Lycopersicon esculentum* leaves was not hepatotoxic in rats. This result is in accordance with those of Weremfo et al., 2011, which showed that the pulp of tomato possess hepatoprotective activity against Ccl4 (carbon tetrachloride) that induced liver damage [40]. Similarly, this result corroborates that of Uchendu (2018), which showed that oral administration of tomato fruit extract significantly protected the liver of albino rats from severe hepatic damage [41]. This hepatoprotective effect of the extract is confirmed by histopathological analysis of the liver that showed no lesions at all treatment doses in both males and females. This result is similar to that of Uchendu (2018), which showed hepatoprotective effect of tomato fruit extract against acetaminophen-induced acute hepatotoxicity in rats [41].

In females, HDL cholesterol levels increased at doses of 250 and 500 mg/kg and showed no significant difference at a dose of 1000 mg/kg compared with controls. Whereas, in males, there was no significant difference in HDL cholesterol but rather a decrease in total cholesterol, LDL cholesterol ,and triglycerides at all doses compared with controls. In both sexes, we observed a reduction in the index of atherosclerosis. These could be explained by the fact that the leaves extract contain polyphenols (flavonoids) with antioxidant properties that could reduce the level of bad cholesterol, increase the level of HDL, and prevent the oxidation of fat. This is in agreement with the idea of Asim 2011 [42], which showed that tomato contains several antioxidants, including polyphenols, lycopene, trace elements, and vitamins, that are known to inhibit atherosclerotic processes. Furthermore, clinical evaluation showed that plasma antioxidation and phenolic contents were increased after administration of fresh tomato and tomato juice. TG levels were decreased after administration of fresh tomato and tomato juice. HDL-C was found to increase, whereas LDL-C was found to decrease after their consumption [43].

The role of the kidney in the body cannot be overemphasized. Like the liver, the kidney is also a major organ that functions in maintaining a steady state in the body. It also excretes waste product of metabolism, including drugs and their metabolites. However, exposure of the kidney to some of these toxic substances may damage the renal tubules [44]. Despite their limitations, high serum creatinine, blood urea nitrogen, and low urine output are currently used in diagnosis of acute renal injury [45]. In the present study, we observed in female rats a decrease of serum urea levels at doses of 250 and 500 mg/kg but serum urea increase at a dose of 1000 mg/kg. In males, the increase of creatinine levels was not significant at all doses, and serum urea levels declined at doses of 250 and 500 mg/kg; but serum urea was always significantly increased at a dose of 1000 mg/kg. This reduction can be explained by the fact that the secondary metabolites contained in the extract are responsible for the reduction of creatinine and urea serum. This reduction is in harmony with the idea of Lee et al. (2016) that showed that the administration of tomato leaf extracts reduced significantly the level of creatinine and urea. This reduction could be a result of the antioxidant effect of flavonoids [46]. Adel et al. (2016) showed that flavonoids upregulate antioxidant defenses and reduce free radical formation, hence exhibiting their powerful antioxidant activity [47]. The enhancement of serum urea at a dose of 1000 mg/kg is believed to be due to the increased absorption of macromolecules at the intestinal tract caused by the ingestion of the tomatine contained in the extract. When it is orally ingested, the brush border of the intestine is damaged by the membrane-disruptive properties of this component, so increased uptake of macromolecules occurs. This damage to the epithelial barriers is dose dependent [25,48]. In vitro studies showed that this glycoalkaloid increases the permeability of the small intestinal mucosal cell, resulting in the inhibition of active nutrient transport and facilitation of the uptake of gut contents that normally would not be absorbed [13, 49].

Increased levels of white blood cells, lymphocytes, and platelets at all doses in males compared with controls may be due to the immunostimulatory effects of the chemical compounds in the extract. This result corroborates those of Sandhu et al. [23], which showed that oral immunization of mice with ripe transgenic tomato fruits led to the induction of both serum and mucosal RSV-F (Respiratory syncytial virus-fusion) specific antibodies [14]. In addition, tomatine affects cytokine-mediated genes, suggesting that it might have immunomodulatory properties [25]. This immunostimulating effect is confirmed by histopathological studies of the liver, which showed leukocytic infiltrations.

## 5. Conclusion

The aim of this research was to evaluate the acute and subacute oral toxicity of this methanolic extract of *L. esculentum* L. leaves. No acute toxicity was found in rats treated with this extract, and approximate lethal dose 50 was determined to be higher than 5000 mg/kg. This methanolic leaves extract of this plant was not hepatotoxic after long-term treatment in both sexes at all doses. In females, this extract promotes the increase of HDL cholesterol levels at doses of 250 and 500 mg/kg; in male rats, it promotes the decrease of total cholesterol and triglycerides at all doses compared with controls. This study shows a lower toxic effect of extract at a dose of 1000 mg/kg with prolonged use (28 days), with proven immunostimulatory effects. Thus, prolonged use of this extract in lower doses (250 and 500 mg/kg) might be advisable, and highest dose (1000 mg/kg) should be avoided.

## Figures and Tables

**Table 1:** Body weights (g) of female rats treated with methanolic extract of *Lycopersicon esculentum.*

Period (days)	Body weights of female rats (g)		
	Female 1	Female 2	Female 3
1^st^ day	180	175	189
15^th^ day	19	189	199

**Table 2:** Relative organ weights (g) of the female rats in acute toxicity of methanolic extract of *L. esculentum.*

Organs	Organ weight of female rats (g)		
	Female 1	Female 2	Female 3
Liver	3.54	3.19	3.38
Kidneys	0.61	0.63	0.64
Lung	0.64	0.64	0.65
Heart	0.28	0.29	0.29
Spleen	0.39	0.42	0.4

**Table 3:** Organ weights (g) of the female and male rats in subacute toxicity of methanolic extract of *L. esculentum.*

Sexes	Organs	Control	*L. esculentum* extract doses (mg/kg)		
			250	500	1000
Female	Liver (g)	3.56 ± 0.55^a^	3.16 ± 0.12^a^	3.03 ± 0.14^a^	3.30 ± 0.26^a^
	Kidneys (g)	0.70 ± 0.02^a^	0.69 ± 0.03^a^	0.61 ± 0.07^a^	0.68 ± 0.08^a^
	Spleen (g)	0.54 ± 0.1^a^	0.45 ± 0.07^a^	0.39 ± 0.03^a^	0.49 ± 0.10^a^
	Lung (g)	0.70 ± 0.16^a^	0.74 ± 0.10^a^	0.72 ± 0.08^a^	0.75 ± 0.13^a^
	Heart (g)	0.35 ± 0.03^a^	0.32 ± 0.01^a^	0.34 ± 0.03^a^	0.33 ± 0.01^a^

Male	Liver (g)	3.10 ± 0.10^a^	3.05 ± 0.26^a^	3.07 ± 0.12^a^	3.08 ± 0.08^a^
	Kidneys (g)	0.64 ± 0.05^a^	0.63 ± 0.05^a^	0.69 ± 0.06^a^	0.68 ± 0.00^a^
	Spleen (g)	0.46 ± 0.08^a^	0.35 ± 0.01^a^	0.46 ± 0.09^a^	0.42 ± 0.04^a^
	Lung (g)	0.73 ± 0.08^a^	0.58 ± 0.03^a^	0.64 ± 0.07^a^	0.78 ± 0.18^a^
	Heart (g)	0.32 ± 0.05^a^	0.31 ± 0.02^a^	0.31 ± 0.00^a^	0.31 ± 0.00^a^

Values are presented as mean ± standard deviation of 4 repetitions. In the same line and by sex, the values bearing the different letters are significantly different (*p* < 0.05).

**Table 4:** Biochemical parameters (ALAT and ASAT) in the serum of female and male rats orally treated with methanolic extract of *L. esculentum*.

Sexes	Parameters	Control	*L. Esculentum* extract doses (mg/kg)		
			250	500	1000
Female	ALAT (U/I)	27.06 ± 4.96^ab^	22.26 ± 2.89^a^	32.30 ± 5.91^b^	22.11 ± 3.51^a^
	ASAT (U/I)	38.41 ± 2.78^b^	27.06 ± 3.55^a^	42.77 ± 5.05^b^	30.84 ± 3.77^a^
	T. proteins (g/dL)	5.10 ± 0.15^a^	5.42 ± 0.33^a^	5.09 ± 0.56^a^	5.06 ± 0.29^a^

Male	ALAT (U/I)	35.39 ± 2.69^b^	28.80 ± 1.56^a^	28.15 ± 2.62^a^	26.62 ± 1.56^a^
	ASAT (U/I)	49.85 ± 4.95^b^	41.90 ± 3.05^a^	38.41 ± 1.96^a^	43.21 ± 2.89^a^
	T. proteins (g/dL)	3.91 ± 0.09^a^	3.88 ± 0.23^a^	3.56 ± 0.14^a^	4.68 ± 0.16^b^

Values are presented as mean ± standard deviation of 4 repetitions. In the same line and by sex, the values bearing the different letters are significantly different (*p* < 0.05). ALAT: alanine aminotransferase; ASAT: aspartate aminotransaminase; T. proteins: total proteins.

**Table 5:** Effect of *L. esculentum* extraction on the level of serum creatinine and serum urea.

Sexes	Parameters (mg/dL)	Control	*L. esculentum* extract doses (mg/kg)		
			250	500	1000
Female	Creatinine	1.03 ± 0.23^a^	0.69 ± 0.17^a^	0.86 ± 0.48^a^	1.01 ± 0.37^a^
	Urea	103.47 ± 3.96^b^	70.13 ± 3.96^a^	103.24 ± 6.41^b^	184.72 ± 3.65^c^

Male	Creatinine	0.66 ± 0.11^a^	0.88 ± 0.13^a^	0.77 ± 0.23^a^	0.95 ± 0.16^a^
	Urea	70.83 ± 5.21^a^	75.00 ± 7.54^a^	73.14 ± 6.46^a^	138.88 ± 5.88^b^

Values are presented as mean ± standard deviation of 4 repetitions. In the same line and by sex, the values bearing the different letters are significantly different (*p* < 0.05).

**Table 6:** Effect of administration of methanolic extract of the leaves of *L. esculentum* on lipid profile in both female and male rats.

Sexes	Parameters	Control	*L. esculentum* extract doses (mg/kg)		
			250	500	1000
Female	TG (mg/dL)	61.25 ± 3.40^ab^	56.84 ± 1.76^a^	64.88 ± 2.44^b^	58.63 ± 7.23^ab^
	TC (mg/dL)	56.37 ± 2.16^a^	76.47 ± 2.20^b^	60.17 ± 3.63^a^	58.16 ± 2.72^a^
	HDL (mg/dL)	39.55 ± 2.16^a^	55.67 ± 3.55^c^	45.33 ± 0.79^b^	39.10 ± 2.16^a^
	LDL (mg/dL)	4.52 ± 0.81^b^	9.42 ± 1.45^d^	1.85 ± 0.10^a^	7.33 ± 0.94^c^
	AI	0.42 ± 0.02^b^	0.37 ± 0.05^a^	0.32 ± 0.02^a^	0.48 ± 0.03^c^

Male	TG (mg/dL)	129.56 ± 8.40^c^	111.77 ± 3.36^b^	110.56 ± 6.24^b^	93.45 ± 1.45^a^
	TC (mg/dL)	85.66 ± 2.50^b^	72.79 ± 3.48^a^	76.59 ± 2.50^a^	77.08 ± 2.70^a^
	HDL(mg/dL)	46.76 ± 2.83^b^	44.62 ± 2.05^ab^	41.49 ± 2.44^a^	45.74 ± 1.60^b^
	LDL (mg/dL)	12.98 ± 1.56^b^	5.81 ± 0.52^a^	12.98 ± 1.47^b^	12.64 ± 1.38^b^
	AI	0.88 ± 0.04^b^	0.63 ± 0.05^a^	0.84 ± 0.06^b^	0.68 ± 0.07^a^

Values are presented as mean ± standard deviation of 4 repetitions. In the same line and by sex, the values bearing the different letters are significantly different (*p* < 0.05). TG: triglyceride; (TC): total cholesterol; HDL: high-density lipoproteins; AI: atherosclerosis-index.

**Table 7:** Hematological parameters of female and male rats.

Sexes	Parameters	Control	*L. esculentum* extract doses (mg/kg)		
			250	500	1000
Female	WBCs(X 10^3^/*μ*l)	14.10 ± 0.60^a^	12.23 ± 1.48^a^	14.46 ± 1.42^a^	13.96 ± 1.45^a^
	Lymph (%)	83.50 ± 4.21^b^	70.30 ± 4.90^a^	76.80 ± 6.87^ab^	77.06 ± 6.90^ab^
	MONO (%)	7.63 ± 0.94^ab^	9.35 ± 1.95^b^	5.73 ± 0.60^a^	6.60 ± 0.26^a^
	PLT (X 10^3^/*μ*l)	677.33 ± 30.07^d^	342.63 ± 38.01^a^	592.66 ± 20.03^c^	466.66 ± 31.06^b^
	MPV (fL)	11.03 ± 1.36^a^	10.46 ± 1.50^a^	10.56 ± 1.62^a^	11.83 ± 1.12^a^
	RBCs(X 10^6^/*μ*l)	7.29 ± 0.20^a^	7.60 ± 1.14^a^	7.73 ± 1.71^a^	6.92 ± 0.18^a^
	Hb (g/dL)	16.60 ± 0.70^a^	15.66 ± 0.15^a^	15.26 ± 0.83^a^	15.63 ± 0.68^a^
	HCT (%)	45.63 ± 3.82^a^	45.73 ± 7.07^a^	46.20 ± 9.92^a^	42.00 ± 1.83^a^
	MCV (fL)	62.60 ± 3.98^a^	60.20 ± 0.75^a^	59.46 ± 1.13^a^	60.73 ± 1.49^a^
	MCH (pg)	22.70 ± 0.88^a^	20.83 ± 2.80^a^	20.06 ± 3.52^a^	22.56 ± 0.60^a^
	MCHC (g/dL)	36.40 ± 1.76^a^	34.70 ± 4.87^a^	33.76 ± 5.45^a^	37.16 ± 0.15^a^

Male	WBCs(X 10^3^/*μ*l)	10.60 ± 1.01^a^	15.96 ± 0.64^b^	17.86 ± 1.79^bc^	19.50 ± 0.80^c^
	Lymph (%)	54.96 ± 2.23^a^	68.36 ± 2.15^b^	70.43 ± 5.70^b^	73.66 ± 2.19^b^
	MONO (%)	8.36 ± 0.68^a^	8.90 ± 0.75^ab^	10.06 ± 0 .40^b^	9.15 ± 0.45^ab^
	PLT (X 10^3^/*μ*l)	334.33 ± 44.81^a^	429.33 ± 35.64^b^	397.66 ± 8.73^ab^	450.00 ± 34.11^b^
	MPV (fL)	9.70 ± 2.26^a^	9.56 ± 0.40^a^	8.70 ± 0.30^a^	9.83 ± 1.55^a^
	RBCs(X 10^6^/*μ*l)	6.34 ± 1.93^a^	6.91 ± 0.32^a^	6.71 ± 1.03^a^	7.86 ± 0.58^a^
	Hb (g/dL)	15.43 ± 1.41^a^	15.46 ± 0.66^a^	13.80 ± 3.40^a^	17.06 ± 1.01^a^
	HCT (%)	39.20 ± 7.07^a^	39.00 ± 2.95^a^	39.76 ± 7.15^a^	44.66 ± 3.66^a^
	MCV (fL)	63.90 ± 10.23^a^	56.43 ± 3.36^a^	59.16 ± 2.56^a^	56.83 ± 0.80^a^
	MCH (pg)	25.66 ± 6.79^a^	22.30 ± 1.12^a^	20.43 ± 3.37^a^	21.70 ± 0.75^a^
	MCHC (g/dL)	39.80 ± 4.01^a^	39.66 ± 1.50^a^	34.53 ± 4.28^a^	38.20 ± 1.30^a^

Values are presented as mean ± standard deviation of 4 repetitions. In the same line and by sex, the values bearing the different letters are significantly different (*p* < 0.05). WBCs: white blood cells, Lymph: lymphocytes, Mono: monocytes, PLT: platelets, MPV: mean platelet volume, RBCs: red blood cells, Hb: hemoglobin, HCT: hematocrit, MCV: mean corpuscular volume, MCH: mean corpuscular hemoglobin, MCHC: mean corpuscular hemoglobin concentration.

## Data Availability

All data generated or analyzed during this study are included in this published article.
